# Biodiversity of Coleoptera (Insecta) in Mordovia State Nature Reserve (Russia) using fermental traps

**DOI:** 10.3897/BDJ.10.e96989

**Published:** 2022-12-19

**Authors:** Leonid Egorov, Alexander Ruchin, Mikhail Esin, Oleg Artaev

**Affiliations:** 1 Prisursky State Nature Reserve, Cheboksary, Russia Prisursky State Nature Reserve Cheboksary Russia; 2 Joint Directorate of the Mordovia State Nature Reserve and National Park «Smolny», Saransk, Russia Joint Directorate of the Mordovia State Nature Reserve and National Park «Smolny» Saransk Russia; 3 Papanin Institute for Biology of Inland Waters Russian Academy of Sciences, Borok, Russia Papanin Institute for Biology of Inland Waters Russian Academy of Sciences Borok Russia

## Abstract

**Background:**

Protected areas are unique ecosystems that are minimally affected by anthropogenic activities. Therefore, in many cases, they are refugia and relevance of faunistic research is undeniable here. A simple method of catching insects, such as trapping with the help of fermental traps, was used in this area for the first time. The authors of the dataset used this method from 2018 to 2021. One thousand and fifty-one traps of our own design were installed. The dataset includes data on 367 species from 52 families (6,497 records of 44,600 specimens). Ten species were dominant in the traps (*Cryptarchastrigata*, *Protaetiamarmorata*, *Glischrochilusgrandis*, *Glischrochilushortensis*, *Soroniagrisea*, *Rhagiummordax*, *Lepturathoracica*, *Lepturaquadrifasciata*, *Quediusdilatatus* and *Protaetiafieberi*). These species accounted for 76.9% of all individuals in the total amount of captured specimens. *Cryptarchastrigata* was the most numerous species (28.8% of the total) and the most frequently encountered species (64.9%). The greatest species diversity was recorded in the families Cerambycidae (53 species), Elateridae and Curculionidae (39 species each), Nitidulidae and Coccinellidae (22 species each). The dataset contains information on the occurrence of 15 rare species.

**New information:**

We have recently published a checklist of the Coleoptera of Mordovia State Nature Reserve (Egorov et al. 2020). It included 2,145 species from 88 families. However, the published list did not contain information about the occurrence of various species of beetles, especially caught in recent years. Part of this list contained information about species collected using fermental traps. However, the list of species did not provide information about specific locations.

## Introduction

There are many reasons that cause the changes in ecosystems. Urbanisation, toxic chemical pollution, regular fires, deforestation and climate change have recently had a significant impact on biodiversity (Myers and Knoll 2001, Lambin et al. 2003, Ruchin et al. 2019, Cicort-Lucaciu 2020, Dedyukhin 2020, Kozak et al. 2020, Shinkarenko et al. 2021). However, in many parts of the world, many ecosystems have not been touched by humans. Such ecosystems are a biodiversity hotspot and they usually have the status of protected areas (Ruchin and Khapugin 2019, Vieira et al. 2019, Bondarenko et al. 2020, Mohd-Azlan 2020). The traditional study of fauna is based on the use of several techniques. The most common ways of studying biodiversity are trapping with various nets, pitfall traps, light traps and pan traps (Vrdoljak and Samways 2012, Alexeev and Aleksanov 2017, Barkalov and Khruleva 2021). Less often, scientists use Malaise traps, flight interception traps, cow manure-baited pitfall traps, rodent burrow pitfall traps, green Lindgren funnel traps and other methods in their research (Quinto et al. 2013, Skvarla et al. 2020, de Souza Amorim et al. 2022). However, trapping with various baits is an equally effective way to study biodiversity. This method makes it possible to identify species that are very difficult to catch by other methods (Allemand and Aberlenc 1991, Redolfi De Zan et al. 2017, Dvořák et al. 2020).

The purpose of this article is to describe a set of up-to-date data on the occurrence and abundance of Coleoptera in the Mordovia State Nature Reserve that has been recently published in GBIF (Ruchin et al. 2022).

## Sampling methods

### Sampling description

Fermental traps were used to collect insects. The traps are a plastic 1.5 and 5-litre container with a window cut out in it on one side at a distance of 10 cm from the bottom. With the help of a weight, a rope with a tied trap was thrown on to a tree branch at a height of 1.5 to 12 m from the soil surface (Ruchin et al. 2020). As bait, fermenting beer, vinegar, white and red dry wine was used with an addition in the form of honey, jam or sugar (Ruchin and Egorov 2021a, Ruchin and Egorov 2021b).

## Geographic coverage

### Description

Mordovia State Nature Reserve is located in the Republic of Mordovia (Central Russia) and has an area of 321.62 km^2^. Main ecosystems are forests of different types. Forests occupy 89.3% of entire territory. *Pinussylvestris* L. is the main forest species that forms pure or mixed plant communities in the southern, central and western parts. Forests consisting of *Betulapendula* Roth occupy second place in terms of area and they were formed in areas of felled and burnt pine forests. A lot of young birch forests are located in places damaged by a forest fire in 2010 (Ruchin et al. 2019). In 2021, the same areas were damaged by fires as in 2010. Therefore, in some places of the Mordovia State Nature Reserve, completely burnt-out areas have appeared. Deciduous forests of *Quercusrobur* L. and *Tiliacordata* are located mainly in northern, western and south-western parts. They are common in the floodplain of the Moksha, Satis and Arga rivers. Forests are dominated by *Piceaabies* L. and *Alnusglutinosa* (L.) Gaertn. They are located mainly in floodplains of small rivers and streams and occupy small areas (Khapugin et al. 2016, Ruchin and Antropov 2019).

### Coordinates

54°42'24"N and 54°56'08"N Latitude; 43°37'49"E and 43°04'28"E Longitude.

## Taxonomic coverage

### Description

Classification of the family-group taxa used in this checklist follows predominantly Bouchard (2011), with subsequent additions (Bouchard and Bousquet 2020). Changes have been taken into account from the Catalog of Palaearctic Coleoptera (Löbl and Smetana 2011, Löbl and Smetana 2013, Löbl and Löbl 2015, Löbl and Löbl 2016, Löbl and Löbl 2017, Danilevsky 2020, Iwan and Löbl 2020), as well as on Cucujoidea from the article by Robertson (2015) and on Curculionoidea from the publication by Alonso-Zarazaga (2017). To clarify the nomenclature, the cited works were used, as well as the Catalog of Palaearctic Coleoptera (Löbl and Smetana 2007, Löbl and Smetana 2010). The years of description of some species are specified according to Bousquet (2016).

## Usage licence

### Usage licence

Creative Commons Public Domain Waiver (CC-Zero)

## Data resources

### Data package title

Coleoptera of the Mordovia State Nature Reserve: study using fermental traps

### Resource link

https://www.gbif.org/dataset/6194f318-adeb-4dea-970e-74707572cb81

### Alternative identifiers

https://doi.org/10.15468/aszj7a

### Number of data sets

1

### Data set 1.

#### Data set name

Coleoptera of the Mordovia State Nature Reserve: study using fermental traps

#### Description

The dataset are described here.

**Data set 1.** 

Column label	Column description
eventID	An identifier for the set of information associated with an Event (occurs in one place in one time).
occurrenceID	An identifier for the Occurrence (as opposed to a particular digital record of the occurrence).
basisOfRecord	The specific nature of the data record: Human Observation.
scientificName	The full scientific name including the genus name and the lowest level of taxonomic rank with the authority.
kingdom	The full scientific name of the kingdom in which the taxon is classified.
taxonRank	The taxonomic rank of the most specific name in the scientificName.
decimalLatitude	The geographic latitude of location in decimal degree.
decimalLongitude	The geographic longitude of location in decimal degrees.
geodeticDatum	The ellipsoid, geodetic datum or spatial reference system (SRS) upon which the geographic coordinates given in decimalLatitude and decimalLongitude are based.
countryCode	The standard code for the country in which the Location occurs.
individualCount	The number of individuals represented present at the time of the Occurrence.
eventDate	The date when material from the trap was collected or the range of dates during which the trap collected material.
year	The integer day of the month on which the Event occurred.
month	The ordinal month in which the Event occurred.
day	The integer day of the month on which the Event occurred.
samplingProtocol	The names of, references to, or descriptions of the methods or protocols used during an Event.
recordedBy	A person, group or organisation responsible for recording the original Occurrence.
sampleSizeValue	A numeric value for a measurement of the size of a sample in a sampling event.
sampleSizeUnit	The unit of measurement of the size of a sample in a sampling event.
samplingEffort	The amount of effort expended during an Event (exposure time, number of days the trap was set).
bibliographicCitation	A bibliographic reference for the description of the methodology.

## Additional information

A total of 6,497 records on Coleoptera occurrence have been published from the territory of Mordovia State Nature Reserve (Russian Federation). The dataset includes data on 367 Coleoptera species from 52 families (44,600 specimens). Ten species were dominant in the traps (*Cryptarchastrigata*, *Protaetiamarmorata*, *Glischrochilusgrandis*, *Glischrochilushortensis*, *Soroniagrisea*, *Rhagiummordax*, *Lepturathoracica*, *Lepturaquadrifasciata*, *Quediusdilatatus* and *Protaetiafieberi*). These species accounted for 76.9% of all individuals in the total amount of captured specimens. *Cryptarchastrigata* was the most numerous species (28.8% of the total) and the most frequently encountered species (64.9%). The largest number of species that were found in traps belongs to the family Cerambycidae (53 species), Elateridae and Curculionidae (39 species each), Nitidulidae and Coccinellidae (22 species each) (Table 1). However, the number of species differed by the year of research. Maximum species diversity and abundance of these families was obtained in 2020 with the installation of the largest number of traps. The dataset contains information on the findings of 15 Coleoptera rare species. Our results showed that an increase in number of traps is not as effective within the third and fourth years of the study compared to the first two years.

**Figure 1.**
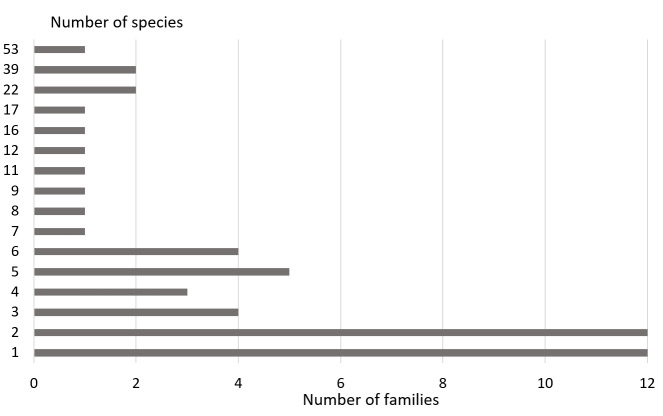
Distribution of families by the number of captured species on the territory of Mordovia State Nature Reserve.

At the same time, the overwhelming number of families were represented in our catches by single species and specimens (Fig. 1). Only one species was recorded in 12 families, and only two species in 12 families. Apparently, the main number of species of most of these families accidently end up in traps and the bait does not serve as an attractant for them.

**Figure 2.**
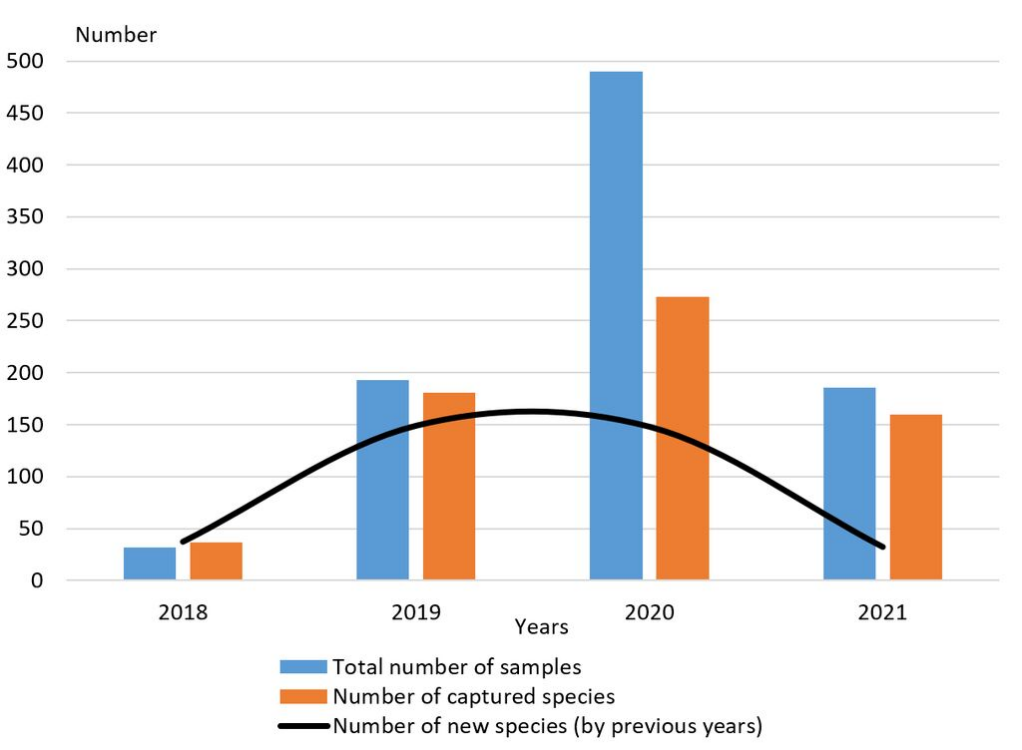
Dependence of the number of captured species on the number of traps by year.

As studies have shown (Fig. 2), an increase in the number of traps in the third and fourth years of study is not as effective as in the first two years. From 2018 to 2021, we increased the number of traps set to study Coleoptera biodiversity using this method and also installed these traps in different biotopes, at different heights and for the entire growing season (Ruchin 2021, Ruchin et al. 2021a, Ruchin and Egorov 2021b). It turned out that the number of species that fall into traps increased significantly in the second year of research with an increase in the number of traps. However, in the third year of research, despite a more significant number of traps, the number of new species that had not previously fallen into such traps did not increase. At the same time, in the 4^th^ year of research, the number of new species not previously caught decreased by 5 times (number of trap exposures decreased only 2.6 times). Random and/or very rare species that live in a particular biotope already fall into the traps. Earlier (Ruchin et al. 2021b), it was suggested that two-year studies would be sufficient to study the biodiversity of a certain biotope or a small region. In this study, at the level of a small territory, we confirm this assumption.

**Figure 3.**
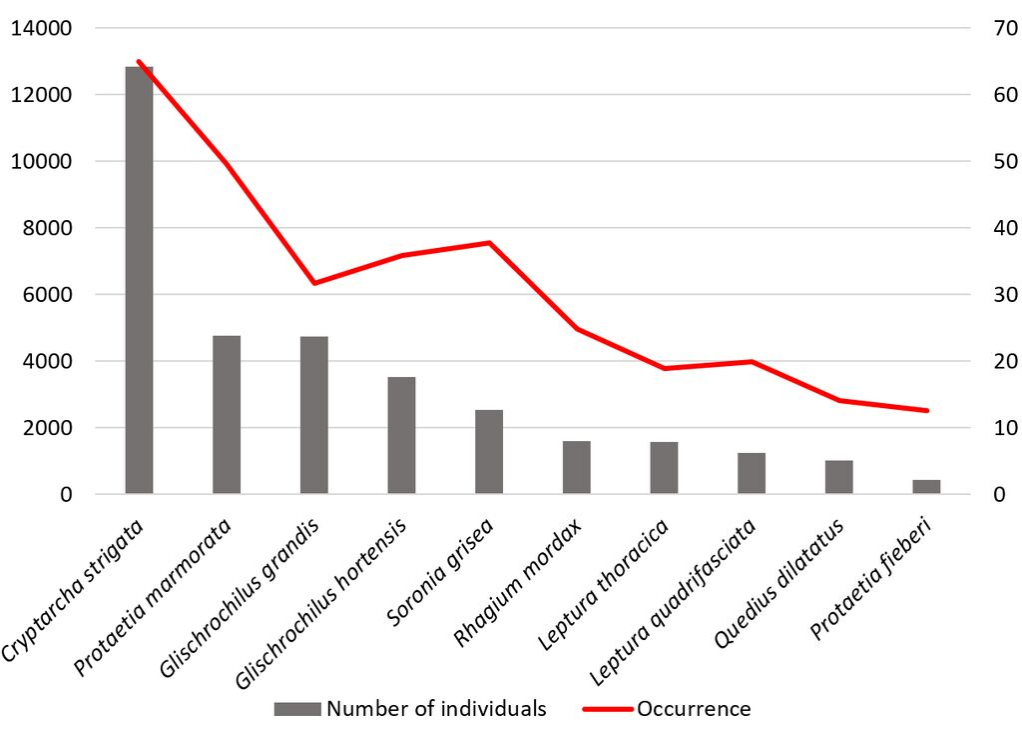
Total number of individuals and occurrence of main dominant species in traps (for the entire period of research).

Despite a significant number of families and a large species diversity, there are several species that are quite common in traps and well lured by fermentation products. We identified 10 species from four families, for which the numerical abundance and occurrence were the greatest in our studies (Fig. 3). These species accounted for 76.9% of all individuals by total amount of captured specimens. *Cryptarchastrigata* was the most numerous species (28.8% of the total) and the most frequently encountered species (64.9%). For 4 years, the number of *Protaetiamarmorata* and *Glischrochilusgrandis* in traps was almost the same (10.7% each). However, occurrence of the first species was 49.7%, while occurrence of the second one was 31.7%. Thus, the vast majority of species were found in traps much less frequently (no more than 10% of the number of traps) and in a very small number of specimens (no more than 1% of the total number of individuals).

During the research period, new information was obtained about species that are listed in the Red Book of Russia (Pavlov 2021) and the Red Book of the Republic of Mordovia (Astradamov 2005). As a result, new localities became known for 13 protected species. Four species (*Protaetiafieberi*, *Protaetiaspeciosissima*, *Osmodermabarnabita* and *Elaterferrugineus*) were included in the Red Book of Russia (Table 2). The high number of observations, abundance and occurrence of *Protaetiafieberi* attracts attention. The species *Protaetiaspeciosissima*, *Osmodermabarnabita* and *Elaterferrugineus* are quite rare.

It is necessary to mention the findings of two more rare species of Coleoptera that are not included in any of the above Red Books. These are *Allonyxquadrimaculatus* (Schaller, 1783) and *Lepturaaurulenta* Fabricius, 1793. Registration of the first species is only the fourth finding of the species on the territory of Russia (the second in a row on the territory of the Mordovia State Nature Reserve) (Ruchin and Egorov 2018a). Two specimens were found in two habitats. *L.aurulenta* is also a very rare species, which was once found on the territory of Mordovia State Nature Reserve in a floodplain deciduous forest (Ruchin and Egorov 2018b). In traps, this species is found in three other habitats, which are also broad-leaved forests. Apparently, populations of both of these species are quite stable, although they are few in the studied territory. The species is distributed in Mordovia on the eastern border of its range.

## Figures and Tables

**Table 1.** Species richness of Coleoptera collected using fermental traps on the territory of Mordovia State Nature Reserve.

Family	Number of species				Number of specimens				TOTAL	
	2018	2019	2020	2021	2018	2019	2020	2021	specimens	species
Cerambycidae	12	37	40	27	143	1652	1948	1790	5533	53
Elateridae	1	23	33	14	2	77	313	127	519	39
Curculionidae	3	20	21	9	3	502	1430	477	2412	39
Nitidulidae	8	13	21	20	169	3768	12626	10722	27285	22
Coccinellidae	0	8	20	7	0	20	61	36	117	22
Cantharidae	1	5	12	4	1	14	112	13	140	17
Carabidae	0	5	11	2	0	7	38	2	47	16
Chrysomelidae	0	2	9	3	0	4	25	4	33	12
Scarabaeidae	6	9	9	8	508	1621	2875	925	5929	11
Histeridae	0	3	8	5	0	21	37	149	207	9
Tenebrionidae	0	4	5	4	0	21	15	6	42	8
Buprestidae	0	4	3	2	0	5	4	3	12	7
Dermestidae	1	7	4	6	1	265	53	308	627	7
Silphidae	4	4	5	3	26	29	137	39	231	6
Melyridae	0	3	5	5	0	10	96	9	115	6
Monotomidae	0	1	5	3	0	15	18	28	61	6
Melandryidae	0	0	5	2	0	0	7	5	12	6
Scirtidae	0	3	3	4	0	8	3	4	15	5
Ptinidae	0	3	3	0	0	8	3	0	10	5
Cleridae	0	5	3	3	0	12	5	15	32	5
Oedemeridae	0	0	4	4	0	0	17	12	29	5
Anthribidae	0	1	5	2	0	2	12	3	17	5
Latridiidae	0	3	2	0	0	3	2	0	5	4
Mycetophagidae	0	2	4	3	0	18	22	17	57	4
Erotylidae	0	1	3	2	0	2	7	3	12	3
Silvanidae	0	1	1	1	0	1	1	1	3	3
Mordellidae	0	1	3	2	0	1	9	7	17	3
Scraptiidae	0	1	2	1	0	1	2	1	4	3
Leiodidae	0	0	2	0	0	0	2	0	2	2
Staphylinidae	1	1	1	2	6	286	693	45	1030	2
Lucanidae	0	1	2	0	0	1	2	0	3	2
Eucnemidae	0	1	1	0	0	1	1	0	2	2
Lycidae	0	1	1	2	0	1	1	2	4	2
Cucujidae	0	1	2	1	0	1	2	1	4	2
Cerylonidae	0	2	0	0	0	2	0	0	2	2
Ciidae	0	0	1	1	0	0	1	1	2	2
Zopheridae	0	0	2	1	0	0	2	1	3	2
Pyrochroidae	0	2	2	1	0	2	2	1	5	2
Anthicidae	0	0	2	1	0	0	2	1	3	2
Brentidae	0	1	0	1	0	1	0	1	2	2
Dytiscidae	0	1	0	0	0	1	0	0	1	1
Hydrophilidae	0	0	1	0	0	0	1	0	1	1
Hydrochidae	0	1	1	0	0	1	1	0	2	1
Byrrhidae	0	0	1	0	0	0	1	0	1	1
Lampyridae	0	0	1	1	0	0	1	1	2	1
Lymexylidae	0	0	1	0	0	0	1	0	1	1
Endomychidae	0	0	0	1	0	0	0	1	1	1
Boridae	0	0	1	0	0	0	1	0	1	1
Pythidae	0	0	0	1	0	0	0	1	1	1
Salpingidae	0	0	1	0	0	0	1	0	1	1
Orsodacnidae	0	0	1	0	0	0	1	0	1	1
Nemonychidae	0	0	0	1	0	0	0	1	1	1
TOTAL	37	181	273	160	859	8384	20594	14763	44600	367

**Table 2.** Number of observations and occurrence of rare species listed in the Red Data Book of Russian Federation (indicated as *) and the Red Data Book of Republic of Mordovia (indicated as **).

**Taxa**	**Number of** **observations**	**Number of** **specimens**	**Occurrence**, %
*Calosomainquisitor* (Linnaeus, 1758)**	3	10	0.3
*Calathusfuscipes* (Goeze, 1777)**	1	1	0.1
*Dendroxenaquadrimaculata* (Scopoli, 1771)**	16	21	1.5
*Protaetiafieberi* (Kraatz, 1880)*	131	429	12.5
*Protaetiaspeciosissima* (Scopoli, 1786)*	20	32	1.9
*Osmodermabarnabita* Motschulsky, 1845*	3	7	0.3
*Gnorimusvariabilis* (Linnaeus, 1758)**	41	85	3.9
*Elaterferrugineus* Linnaeus, 1758*	11	11	1.0
*Coccinellaquinquepunctata* Linnaeus, 1758**	1	1	0.1
*Lygistopterussanguineus* (Linnaeus, 1758)**	8	12	0.8
*Notoxusmonoceros* (Linnaeus, 1761)**	5	6	0.5
*Necydalismajor* Linnaeus, 1758**	44	62	4.2
*Purpuricenuskaehleri* (Linnaeus, 1758)**	16	29	1.5
